# Exercise, Arterial Crosstalk-Modulation, and Inflammation in an Aging Population: The ExAMIN AGE Study

**DOI:** 10.3389/fphys.2018.00116

**Published:** 2018-02-21

**Authors:** Lukas Streese, Arne Deiseroth, Juliane Schäfer, Arno Schmidt-Trucksäss, Henner Hanssen

**Affiliations:** Division of Sports and Exercise Medicine, Department of Sport, Exercise and Health, University of Basel, Basel, Switzerland

**Keywords:** aging, vascular function, exercise, arterial stiffness, retinal vessels

## Abstract

**Background:** Age is a key determinant for the development of cardiovascular disease and higher age coincides with an increased prevalence of obesity and physical inactivity. The study examines the influence of physical activity on aging processes of physiological systems focusing on the mechanisms of vascular aging.

**Methods/Design:** The study consists of two parts. The cross-sectional approach aims at examining the association of physical fitness and cardiovascular risk with large and small artery function in healthy older active (HOA, *n* = 40) and sedentary (HOS, *n* = 40) persons as well as older sedentary individuals with increased cardiovascular risk (OSR, *n* = 80) aged 50–80 years. In the interventional approach, the OSR group is randomized into a 12-week walking-based high intensity interval training (HIIT) group or a control condition, aiming at examining the effects of HIIT on arterial function in diseased older adults. Active lifestyle is defined as >9 metabolic equivalent of task (MET) per week and sedentary as ≤3 MET/week. Inclusion criteria for OSR are overweight or obesity (body mass index ≥30 kg/m^2^) plus at least one additional cardiovascular risk factor. The primary outcome is arterial stiffness as determined by aortic pulse wave velocity (PWV). The secondary outcomes are retinal arterial and venous diameters. Further cardiovascular assessments include peripheral PWV, central haemodynamics, retinal endothelial function, carotid intima media thickness, cardiac strain and diastolic function as well as autonomic function and inflammation. Physical fitness is measured by a treadmill-based spiroergometry to determine peak oxygen uptake.

**Discussion:** The aim of the study is to demonstrate the importance of and need for specific physical activity programs for seniors to achieve healthier aging as a long-term goal. Vascular function defines disease- and age-related end organ damage and represents the potential to contain health at older age. This research will identify cardiovascular biomarkers that best resemble underlying cardiovascular risk in age and disease. The integrated approach will help define new recommendations for treatment guidance of exercise therapy in an aging population.

ClinicalTrials. gov: NCT02796976; registered 02 June 2016 (retrospectively registered).

## Background

Atherosclerotic cardiovascular disease (CVD) is a chronic inflammatory disease of the circulatory system and it is a main health care threat in western countries. Almost every third death can be attributed to CVD, amounting to a total of 17.5 million associated deaths worldwide (World Health Organization, 2014). About 80% of CVD deaths are thought to be associated with arterial disorders (Thom et al., 2006). Age is one of the major risk factors for the development of CVD and demographic change further aggravates the enormous socio-economic health care challenge. The number of persons aged 60 or older will double until the year 2050, which will further aggravate the burden of age-related diseases such as CVD (World Population Prospects, 2015). Aging is associated with complex structural and functional alterations of the vascular bed (Ferrari et al., 2003).

The rise of the obesity epidemic in the last decades is another main underlying reason for the high prevalence of CVD. To date, almost 70% of adults are classified as either overweight or obese as compared to 40% 40 years ago (Lavie et al., 2009). The prevalence of obesity and the metabolic syndrome increases with older age (Ford et al., 2004). Physical inactivity is a main risk factor not only for the development of obesity but also for non-communicable diseases in general and CVD specifically. The World Health Organization (WHO) has stated that more than three-quarters of all CVD mortality may be prevented by appropriate changes in lifestyle. Blair et al. found that individuals who improved from unfit to fit over a mean follow-up of 5 years showed a reduction in mortality risk of 44% compared to those who remained unfit (Blair et al., 1995). In a large prospective cohort, Wen et al. reported a 14% reduction of all-cause mortality in individuals exercising for 90 min per week or 15 min per day compared to an inactive group (Wen et al., 2011). Vigorous-intensity exercise (>8.5 MET) seemed to yield greater health benefits in terms of all-cause mortality reduction than moderate-intensity exercise (4–6 MET).

High intensity interval training (HIIT) has been suggested to be an effective training modality for secondary prevention of CVD in older adults and seems to be superior to well-established moderate continuous exercise training with respect to improving not only cardio respiratory fitness but also the cardiovascular risk profile (Helgerud et al., 2007; Wisloff et al., 2007; Tjonna et al., 2008; Guimarães et al., 2010; Kessler et al., 2012; Molmen et al., 2012). The risk of CVD events is considered to be equally low for both HIIT and moderate continuous training (MCT) intervention strategies (Rognmo et al., 2012). Exercise training and regular PA are able to reduce the main underlying mechanisms for the development and progression of CVD such as inflammation, oxidative stress and endothelial dysfunction. However, it is still unclear which biomarker is most suitable to detect the process of vascular aging and can sensitively quantify and monitor treatment effects at older age. Novel approaches for cardiovascular risk screening and exercise treatment strategies are indispensable to counteract the growing socio-economic burden and health hazard of cardiovascular disease in an aging population.

## Cardiovascular health and aging: a systems physiology approach

Vascular aging is a gradual process of the circulation that is aggravated by the development of cardiovascular risk factors and affects both the macro- and microcirculation (Nichols et al., 2011). Since aging is the main denominator for chronic CVD manifestations, the concept of vascular aging has been proposed to improve clinical guidance of patients with increased cardiovascular risk (Nilsson et al., 2009, 2013). The concept implies that age-related clinical or subclinical manifestations are associated with vascular alterations, which can be quantified by sensitive non-invasive vascular assessments. Consistent evidence suggests that arterial stiffness is a subclinical, strong and valid vascular biomarker for the quantification of atherosclerosis and grave cardiovascular dysfunction (Salomaa et al., 1995; Laurent et al., 2006; Vlachopoulos et al., 2010a). Arterial stiffness and the impairment of the buffer capacity of large arteries lead to elevated left ventricular afterload and left ventricular hypertrophy and, at later stages, to heart failure, worsening of coronary artery disease and increased risk of stroke (Hamilton et al., 2007). Aortic pulse wave velocity (PWV), acknowledged as the “gold-standard” method for measuring arterial stiffness, is an independent predictor for cardiovascular morbidity and mortality in the general population, elderly subjects and in patients with cardiovascular disease (Laurent et al., 2001; Sutton-Tyrrell et al., 2005; Hansen et al., 2006; Mattace-Raso et al., 2006). An increase of aortic PWV by 1 m/s has been reported to represent a risk increase of 15% in total cardiovascular and all-cause mortality (Vlachopoulos et al., 2010a). In the Baltimore Longitudinal Study of Aging, aortic PWV increased twofold across the age span and higher fitness were associated with reduced arterial stiffness in a predominantly sedentary population as well as in endurance trained older men compared to less active peers (Vaitkevicius et al., 1993). In a population-based study of 373 younger subjects in the Netherlands, it was found that the effect of habitual PA on arterial stiffness depends on its intensity and differs depending on the arterial tree segment. Vigorous but not light-to-moderate habitual PA provides favorable associations with peripheral arterial stiffness in young adults (van de Laar et al., 2011). In our study, different approaches of measuring arterial stiffness are applied in various vascular beds. Central and peripheral PWV measurements are performed as well as 24-h monitoring of central haemodynamics such as augmentation index (AIx) and pulse pressure (PP). Assessment of the macrocirculation includes the structural analysis of the carotid intima-media thickness (IMT).

It is generally assumed that increased central PWV contributes to the pathogenesis of small vessel disease, particularly the myocardial and cerebral microcirculation. Increased arterial stiffness seems to expose small vessels to highly pulsatile pressure and flow, thereby inducing damage to the microvascular bed (O'Rourke and Safar, 2005). A cross-talk between large and small arteries exists, promoting a vicious circle of increases in peripheral vascular resistance, blood pressure and arterial stiffness, eventually leading to the manifestation of micro- and macrovascular target organ damage (Laurent et al., 2009). Retinal vessel analysis is a non-invasive technique that allows the examination of the retinal microcirculation (Liew et al., 2008). Retinal vessels are part of the cerebrovascular bed and they are affected early in the process of cardiovascular disease. Large cohort studies have previously shown that narrower retinal arterioles, wider retinal venules and a resulting lower arteriolar-to-venular diameter ratio (AVR) are associated with increased risk and severity of hypertension (Wang et al., 2003; Wong et al., 2004a; Ikram et al., 2006b), risk of stroke (Ikram et al., 2006a; McGeechan et al., 2009) and a higher cardiovascular morbidity and mortality in older subjects (Wong et al., 2002; Wang et al., 2007). In older adults, obesity is associated with retinal venular widening and a lower arteriolar-to-venular diameter ratio (AVR) (Nguyen and Wong, 2006; Wang et al., 2006). Inflammation has been associated with wider retinal venular diameters (Klein et al., 2006). We have previously shown that higher physical fitness levels are associated with higher retinal AVR and that regular endurance exercise induced arteriolar dilatation as well as venular constriction, leading to a significantly improved AVR in middle-aged lean and obese individuals (Hanssen et al., 2011). Retinal microvascular endothelial function can directly be measured *in vivo* by dynamic retinal vessel imaging inducing neurovascular coupling by flicker stimulus (Falsini et al., 2002; Polak et al., 2002). An impaired response to flicker light has been associated with type 2 diabetes mellitus (Mandecka et al., 2007; Sörensen et al., 2016), high blood pressure (Nagel et al., 2004) and aging (Pemp et al., 2009; Kotliar et al., 2011). The assessment of retinal vessel diameters is used to define microvascular aging at older age and to examine associations with physical fitness. Retinal vessel analysis allows for the investigation of the cross-talk between large and small arteries.

Large artery stiffness is not only associated with small vessel disease but is known to relate to left ventricular hypertrophy as well as systolic and diastolic dysfunction (Roman et al., 2000; Weber et al., 2008; Namba et al., 2016). In patients with normal ejection fraction, increased pulse wave reflection and PWV are associated with higher left ventricular filling pressures (Weber et al., 2008). In patients with asymptomatic diastolic dysfunction, increased PWV has been shown to precede the onset of manifest heart failure with preserved ejection fraction (Karagodin et al., 2017). Indices of systolic and diastolic function share its predictive character with arterial stiffening (Vlachopoulos et al., 2010b; Kane et al., 2011; Lam et al., 2011). This suggests that ventricular-vascular interactions play a pivotal role in the clinical relevance of arterial stiffness (Chung et al., 2010). The recent Health ABC Study, however, demonstrated that the association of arterial stiffness with the development of heart failure is not independent of cardiovascular risk factors (Pandey et al., 2017). Additional assessment of left ventricular structure and function allows for analysis of the cardiovascular cross-talk and its association with cardiovascular risk factors.

Autonomic function (AF) is a principal regulator of vascular properties and cardiac function. AF alters with age and plays a crucial role in the development of cardiovascular diseases (1996; Fisher et al., 2009). Its sympathetic and parasympathetic branches regulate vascular tone and, thereby, modulate arterial stiffness (Perkins et al., 2006). Heart Rate Variability (HRV) is an easily recordable clinical marker for autonomic function and its indices have shown predictive value for the development of cardiovascular disease (1996; Kleiger et al., 2005). Increased physical activity has been shown to be associated with favorable autonomic function in older adults (Soares-Miranda et al., 2014). In addition to the above mentioned physiological systems, we will measure circulating anti- as well as pro-inflammatory cytokines and analyse their role in mediating physical inactivity- and obesity-related vascular and systemic impairments. Vascular aging and immunosenescence are explained in large part by an imbalance between inflammatory and anti-inflammatory processes. These result in a predominantly pro-inflammatory status that has been termed “inflamm aging” (Franceschi et al., 2007). Healthy aging and longevity are the result of an efficient lifelong anti-inflammatory activity that, once it fails to overcome cellular and systemic inflammatory processes, can be the driving force for frailty and age-related pathologies.

## Methods/design

### Objectives

The aims of the study are twofold. In our cross-sectional research approach, the association of physical activity and fitness on the process of normal (healthy) aging is analyzed by comparing the group of healthy older sedentary (HOS) with healthy older active (HOA) adults. The association of CVD on the process of aging is examined by comparing HOS with older sedentary persons with increased cardiovascular risk (OSR). In the interventional approach, the reversibility of advanced vascular and systemic aging by a walking-based HIIT is examined in OSR.

### Cross-sectional (part I)

Aim 1: To determine the associations of physical fitness and cardiovascular disease with large and small artery function in HOA, HOS as well as OSR group.

### Interventional (part II)

Aim 2: To examine the effects of HIIT on large and small artery function in OSR.

### Outcome measures

Primary outcome: central (carotid-femoral) pulse wave velocity (cfPWV)

Secondary outcomes: central retinal arteriolar (CRAE) and venular (CRVE) diameters

Further outcomes: peripheral (femoral-posterior tibial) pulse wave velocity (ftPWV); central augmentation index (cAIx); central pulse pressure (cPP); 24 h AIx (24AIx) and cPP (24cPP); carotid intima media thickness (IMT); retinal vessel flicker response (DVA); left ventricular diastolic function and myocardial strain; heart rate variability (HRV), inflammation.

### Hypotheses

Hypothesis 1 (Part I): cfPWV is lower in HOA compared to HOS

Hypothesis 2 (Part I): cfPWV is higher in OSR compared to HOS

Hypothesis 3 (Part II): cfPWV in OSR can be reduced by 12 weeks of HIIT compared to controls.

The same hypotheses will be addressed for the secondary and further outcomes.

### Study design

**Figure 1:**
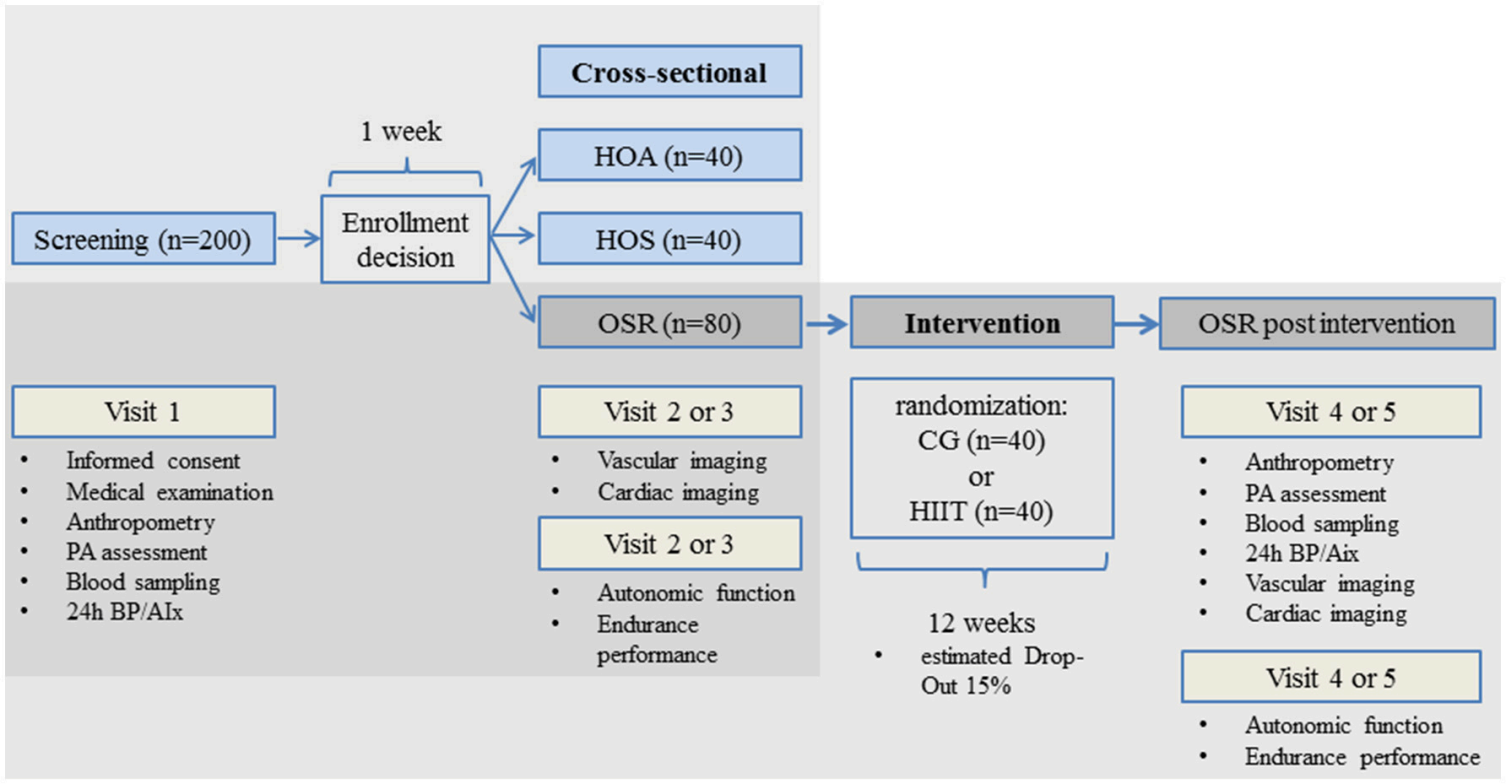
Study design, cross-sectional part (Part I) and randomized controlled trail (RCT) (Part II); HOA, healthy older active; HOS, healthy older sedentary; OSR, older sedentary at risk; CG, control group (standard recommendations for daily physical activity); HIIT, high intensity interval training.

The study design consists of two parts with separate sample size considerations for the cross-sectional and interventional approach. HOA and HOS participants as well as OSR are recruited and enrolled in the cross-sectional study (Figure 1). Recruitment of the OSR group is performed on the basis of agreement to take part in the exercise intervention following the cross-sectional assessment, which serves as a baseline examination for the consecutive intervention. Before enrolment, subjects are medically examined and scanned for inclusion and exclusion criteria by the study physician. Written informed consent is obtained from eligible subjects and a medical examination and anthropometry are performed and physical activity levels are assessed. In addition, blood sampling and 24 h blood pressure monitoring including central PWV, Aix, and PP are undertaken (visit 1). The enrolment decision is taken on the basis of the inclusion and exclusion criteria. On two separate visits and in randomized order, the main vascular diagnostics, echocardiography, autonomic function and the assessment of endurance performance (VO2max) are performed for the cross-sectional part of the study. All vascular diagnostics take place in the morning and under fasting conditions (visit 2 or 3). The endurance performance and the autonomic function is always performed in the afternoon (visit 2 or 3) to reduce within-day variability. Visit 1 and visit 2/3 are separated by at least 1 week to check inclusion/exclusion criteria. Visit 2 and 3 are separated by at least 1 day or no more than 2 weeks. After these three visits, the OSR group is randomized to take part in a 12-week HIIT or a control condition with general lifestyle recommendations. All participants have an equal likelihood of being assigned to treatment or control group. All assessments of the baseline examination are repeated in the follow-up after 12 weeks (Figure 1).

### Inclusion criteria

**Figure 2:**
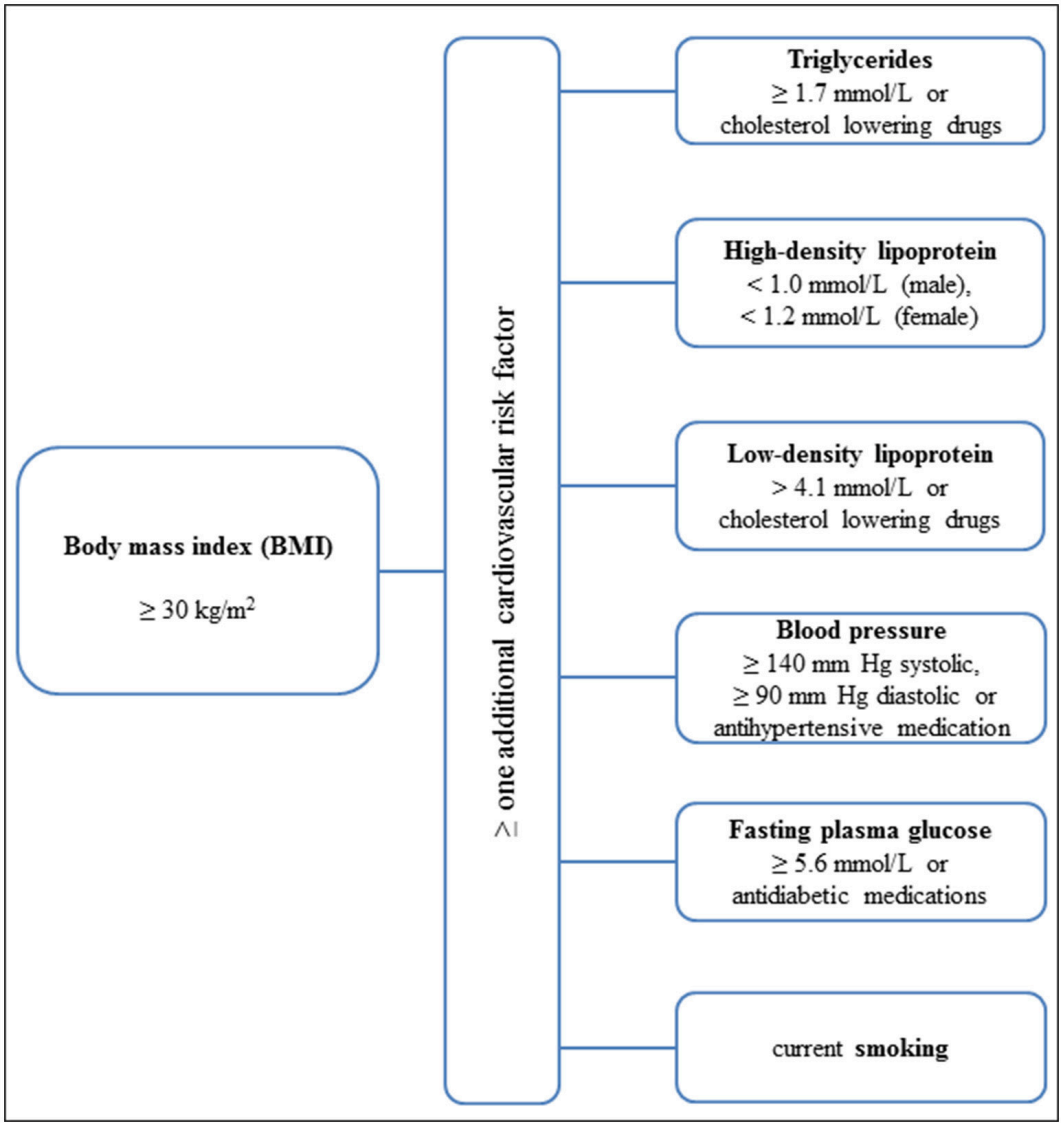
Inclusion criteria and risk factors for older sedentary at risk group (OSR).

- Healthy men and women aged 50–80 years with:
  - active lifestyle: >9 MET/week (>3 h moderate walking/week) or
  - sedentary lifestyle: ≤ 3 MET/week (≤ 1 h moderate walking/week)
  - 18.5 ≤ BMI < 25.0 kg/m^2^
- Sedentary men and women aged 50–80 years with increased cardiovascular risk:
  - overweight or obesity (BMI ≥ 30 kg/m^2^) and
  - ≥one additional cardiovascular risk factor as described in Figure 2.

### Exclusion criteria

- Healthy men and women aged 50–80 years with:

History of cardiovascular, pulmonary or chronic inflammatory disease; blood pressure ≥140/90 mmHg during 24 h monitoring or any of the risk factors in Figure 2; current or past smoker; macular degeneration or glaucoma.

- Sedentary men and women aged 50–80 years at risk:

Decompensated cardiovascular, pulmonary or chronic inflammatory disease; macular degeneration or glaucoma; compromising orthopedic problems.

### Setting

The RCT will be realized at the Department of Sport, Exercise and Health, Basel, Switzerland. This study is financially supported by the Swiss National Science Foundation (SNF) and approved by the Ethics Committee of Northwestern and Central Switzerland (EKNZ-2015-351). We registered this study on ClinicalTrials.gov (NCT02796976) in June 2016.

### Study procedures and ethical considerations

All participants are briefed verbally and receive information approved by the Ethics Committee of Northwestern and Central Switzerland (EKNZ) giving details on the study procedures, the RCT and the process of randomization. The study will be carried out in accordance to the protocol and with the principles stated in the Declaration of Helsinki and the Guidelines of Good Clinical Practice (GCP) (World Medical Association, 2013). This study protocol was design according the SPIRIT Guidelines.

All measurements and procedures applied in this study are non-invasive. Routine exercise ECG includes an exertional stress test as a medical necessity and safety precaution to evaluate cardiorespiratory health in all subjects. The OSR group undergoes a high intensity walking-based training program. HIIT has previously been applied in a wide range of patients including patients with coronary artery disease. The risk for cardiovascular events has been proven to be equally low for both HIIT and moderate continuous intervention strategies (Rognmo et al., 2012). Retinal vessel analysis includes mydriasis of one eye. Using a mydriaticum (Tropicamid 0.5%), the pupils are dilated and enable retinal vessel analysis. The eye drops can cause temporary discomfort, oftentimes a burning sensation for 1–2 min. Flickerlight exposure can potentially cause slight headaches. The collaborating ophthalmology department in Basel will offer back up at all times in the unlikely event of continuous discomfort.

All participants are committed to sign a consent form and will be informed about their right to withdraw from the study without any consequences. A study assistant will randomize the OSR group in an intervention or a control group by drawing a lot after the last pre-intervention assessment. Participants and investigators are blinded for the group membership during the pre-measurements. All participants are to be recruited from our outpatient department, the Metabolism Unit of the University Hospital Basel, Basel, Switzerland and, in addition, with postings on University webpages and advertisements in local newspapers. We aim to recruit the HOA group in running clubs and on running events in and around Basel. A total of 160 participants will be recruited for the study.

### Statistical analysis

The primary outcome of the study is the cfPWV among HOA, HOS, and OSR (part I), and among OSR after 12 weeks of intervention with a high-intensity interval training (HIIT) and those assessed within the same time frame without the HIIT intervention (control group) (part II). To describe continuous demographic and baseline characteristics of participants in the HOA, HOS and OSR group (part I) and those in the OSR intervention and control group (part II), we will use the median and interquartile range; and for categorical characteristics, we will use percentages. Boxplots will be used to visualize the primary and secondary outcomes in the HOA, HOS and OSR group (part I) and at baseline as well as after 12 weeks in the OSR intervention and control group (part II). For part I, we will use analysis of variance to compare the cfPWV (and secondary outcomes) between HOA, HOS and OSR. For part II, we will use analysis of covariance to compare the cfPWV (and secondary outcomes) after 12 weeks between OSR in the intervention and those in the control group adjusted for the corresponding values at baseline (Vickers and Altman, 2001). For each analysis, we will report estimates (with 95% confidence intervals) of the difference in outcome between HOA and HOS and between OSR and HOS (part I), and between OSR in the intervention and those in the control group (part II). Up-to-date versions of SAS (SAS Institute Inc., Cary, NC, USA) and R (R Foundation for Statistical Computing, Vienna, Austria) will be used for analysis and graphics.

### Sample size calculation

The sample size was calculated separately for both study parts. For part I, we assumed that the expected cfPWV corresponded to 8.5, 9.5, and 11.5 m/s for HOA, HOS and OSR, respectively, and that the standard deviation was 1.5 m/s (Tedesco et al., 2004; Gando et al., 2010). With a 2-sided significance level of 0.05, the sample size needed to attain a targeted power of 80% for detecting a difference with magnitude 1.0 m/s was 36 participants per group (the comparison between HOA and HOS) and for detecting a difference with magnitude 2.0 m/s, it was 10 participants per group (the comparison between OSR and HOS). For part II, we assumed that the expected difference in cfPWV after 12 weeks between OSR in the intervention and those in the control group was 1.0 m/s and that the standard deviation was 1.5 m/s (Madden et al., 2009). By including the baseline cfPWV (before the start of the intervention period) as a covariate in the pre-specified analysis, we will further reduce error variability and therefore assumed that the correlation between baseline and outcome cfPWV was 0.3. With a 2-sided significance level of 0.05, the sample needed to attain a targeted power of 80% for showing superiority of the intervention over control was 34 participants per group. Taking dropouts into account, we plan to include a total of 40 HOA, 40 HOS and 80 OSR. The POWER and GLMPOWER procedures in SAS 9.3 (SAS Institute Inc., Cary, NC, USA) were used for sample size calculation for the first and second study part, respectively.

### Exercise intervention and control condition

#### High intensive interval training (HIIT)

The exercise intervention for the OSR intervention group is a supervised Nordic-Walking training three times a week with gradually increased intensity during the first 2 weeks. In the first week, we will teach Nordic Walking technique, warm-up and cool-down elements as well as continuous walking training with moderate intensity at 75% of maximal heart rate (HRmax). In the second week, the intensity will increase to 80–90% of HRmax. In the following 10 weeks, HIIT will be performed as described below. Exercise scientists will supervise all training sessions.

The following protocol will be used (modified from Wisloff et al., 2007):

- Three supervised trainings per week
- Intensity: 10 min warm-up at 65-70% HRmax, 4x4 min interval training at 80-90% HRmax with 3 min of active recovery at 65-70% HRmax, 10 min cool down at 60-70% HRmax
- Duration: 45 min

#### Control group (CG)

The control group is asked to orientate their physical activity according to the European Guidelines on vascular disease prevention in clinical practice (Backer et al., 2003).

## Imaging methods and course of measurements

### Macrovascular aging

#### Central pulse wave velocity

Carotid-femoral PWV (cfPWV), the gold standard for the measurement of arterial stiffness, is assessed by using a SphygmoCor® device (AtCor, Medical Pty Ltd, Sydney, Australia). The good intra- and interobserver reproducibility of this technique has been demonstrated in healthy populations and in patients with chronic kidney disease (Frimodt-Møller et al., 2006, 2008). After 10 min of rest, pulse waves are recorded using a high-fidelity tonometric transducer at two sites (right carotid artery and right femoral artery). Pulse wave travel distance is determined by subtracting the distance between the manubrium to the carotid artery from the distance between the femoral artery to the manubrium as previously described (Townsend et al., 2015). cfPWV measurements are considered to meet the quality control parameters if two consecutive measurements are visually acceptable and within 1 m/s of each other with a standard deviation of <10% (Sigrist et al., 2010). If this criterium could not be reached a third measurement was applied. The mean of all valid measurements represents the cfPWV value.

#### Peripheral pulse wave velocity

Femoral-tibial PWV (ftPWV) is assessed by using the same SphygmoCor® device as for central PWV. Peripheral PWV is determined as the travel time of the pulse wave between the two recording sites at the femoral artery and the posterior tibial artery divided by the distance between the two recording sites. All other procedures and conditions are the same as for central PWV (see above).

#### Pulse wave reflection (PWA)

AIx and AIx@75 are measured using the gold standard device for single measurements (SphygmoCor®). The augmentation index from the central pulse is calculated as: AIx = 100 × (P2 – P1)/pulse pressure, where P2 is the peak of the reflected backward wave, P1 is the peak of the forward pressure wave and central pulse pressure is the systolic pressure maximum minus the diastolic pressure. A positive AIx would indicate an augmentation of peak systolic pressure by the reflected wave. Pulse waveform is obtained using applanation tonometry (SphygmoCor® device, ATCor Medical, Sydney, Australia) on the right radial artery. Ten-second recordings are accumulated until either two analyses with a quality index of ≥90% or three of ≥80% were available. By applying a generalized transfer functions (GTF) the central arterial pulse waveform is estimated by the SphygmoCor® software. These transfer functions have been validated previously during rest (Gallagher et al., 2004) and exercise (Sharman et al., 2006). The calculated central arterial pulse waveform is subjected to further analysis, such as the determination of AIx and AIx@75. Reproducibility has been shown to be acceptable at rest (Filipovský et al., 2000) and during exercise (Holland et al., 2008).

#### 24-h ambulatory monitoring of central hemodynamics

Twenty-four hours ambulatory pulse wave monitoring is obtained using an oscillometric Mobil-O-Graph® 24 h PWA Monitor device (I.E.M GmbH, Germany) with integrated ARCSolver® software. Based on the oscillometric data, central hemodynamics as well as the 24-h PWV is calculated. Oscillometric pulse wave analysis are performed every 20 min for 24 h. Subjects are instructed to hold their arm as steady as possible during the measurements but otherwise maintain their daily routine with no additional physical activity while wearing the device. After data readout, every individual measurement is reviewed for erroneous values. Values are deleted if the quality of data is graded 3 or 4 by the ARCSolver® software. The methods used for these analyses are the same as used by the SphygmoCor® software described previously (Wassertheurer et al., 2010). Validity and reproducibility of oscillometric estimates of cPP, Aix, and AIx@75 are comparable with laboratory settings (Wassertheurer et al., 2010; Luzardo et al., 2012; Papaioannou et al., 2013).

#### Intima media thickness (IMT)

For the semi-automatic evaluation of intima-media thickness (IMT), B-Mode clips are conducted using an ultrasonic device UF-870AG (Fukuda Denshi, Japan) according to current guidelines (Touboul et al., 2012). With the participant in supine position and the head rotated by 45° either to the left or right side two locations on both sides are scanned. Ear-to-ear and horizontal clips are recorded over at least three heart cycles using the US machines inbuilt 3-lead ecg function. Post procession and video-based IMT-admeasurement of the exported clips is performed using the Dynamic Artery Analysis software (DYARA) as described elsewhere (Teynor et al., 2012), using image averaging over three heart cycles. Reproducibility of the aforementioned method is excellent (Touboul et al., 2012).

### Microvascular aging

#### Static retinal vessel analysis (SVA)

The retinal microcirculation is easily accessible by using the Retinal Vessel Analyser (RVA, IMEDOS Systems, Jena, Germany) and a fundus camera (450 FF; Carl Zeiss, Jena, Germany). The technique allows for the analysis of the structure and function of retinal arterioles and venules. To measure retinal vessel diameters, we analyze three valid pictures from one eye with an angle of 50° and the optic disc in the center. The detailed procedure is described elsewhere (Hanssen et al., 2011). Briefly, retinal arterioles and venules, coursing through an area of 0.5–1 disc-diameter from the optic disc margin, will be identified semi-automatically at higher magnification using special analyzing software (Vesselmap 2, Visualis, Imedos Systems UG). Diameters will be averaged to central retinal arteriolar (CRAE) and venular (CRVE) equivalents, and the arteriolar-to-venular-ratio (AVR) will be calculated from the CRAE and CRVE. The inter-observer and intra-observer interclass correlation coefficient for the measurement of retinal vessel diameters ranges from 0.75 to 0.99 (Hubbard et al., 1999; Wong et al., 2004b).

#### Dynamic retinal vessel analysis (DVA)

Functional retinal vessel analysis requires pharmacological dilatation of one pupil with conventional eye drops (Tropicamide 0.5%). Microvascular function is analyzed by a flicker-induced dilatation of retinal arterioles and veins. We use a fundus camera (450 FF; Carl Zeiss, Jena, Germany), with a charge-coupled device for electronic online imaging and a personal computer for system control, analysis and recording of the obtained data, allowing non-invasive assessment of retinal endothelial function mediated by neurovascular coupling following flicker stimulation (Falsini et al., 2002; Polak et al., 2002). The whole procedure is described elsewhere (Nagel et al., 2004; Gugleta et al., 2006; Garhofer et al., 2010). Due to the described inter- and intra-individual variations of vessel diameters (Hubbard et al., 1999), the mean diameter resulting from three baseline measurements before application of the stimulus in each of three flicker cycles is defined as 100%. The ensuing vessel diameter changes are recalculated in % to this individual baseline value (Pemp et al., 2009). From the median of the three curves, parameters such as maximal vessel dilatation, maximal reactive vessel constriction and area under the reaction curve during and after flicker stimulation can be analyzed (Kotliar et al., 2011).

#### Cardiac aging

Echocardiographic parameters are assessed using an ultrasonic device (UF 870AG, Fukuda Denshi, Japan) according to current guidelines (Nagueh et al., 2009; Lang et al., 2015). Briefly, global systolic function is described by ejection fraction based on chamber quantifications as recommended. Linear method and 2D based formulas are used to calculate left ventricular function mass. Right ventricular measurements (such as tricuspid annular plane systolic excursion) as well as atrial volume measurements give further insight into mechanical properties of the heart. Diastolic function is assessed using mitral inflow patterns (E, A, deceleration time, intraventricular relaxation time), tissue Doppler derived mitral annular velocities (E′, A′) and pulmonary artery pressures. Additionally, speckle tracking echocardiography is used to calculate strain.

#### Autonomic aging

We record a 12-lead-ECG for 20 min in supine position before and 10 min after a treadmill test using Custo Diagnostic Software (Custo Med GmbH, Germany). The additional post-treadmill assessment will enable us to evaluate recovery of HRV after acute bouts of exhaustive exercise. Raw data are extracted and processed to derive HRV parameters. Time-domain parameters are standard deviation of normal to normal (NN) RR intervals (SDNN), root mean square of successive differences (RMSSD) and the number of pairs of successive NNs that differ by more than 50 ms (NN50). Frequency-domain paramters include high- and low frequency power. Additionally, Heart Rate Turbulence (HRT) and Deceleration Capacity (DC) are calculated (Schmidt et al., 1999; Bauer et al., 2006).

#### Inflamm-aging and circulating CV risk factors

To analyse the influence of physical activity on immunological processes and the association of inflammation, blood samples are taken. They are drawn by venepuncture of the cubital fossa of the right or left arm by trained medical staff in a fasting state. The blood is transported directly to the clinical chemistry laboratory for further analysis or centrifuged and put on ice. Clinical routine measurements including total blood count, total cholesterol (TC), low- (LDL) and high-density lipoprotein (HDL), triglyzerides (TGA) (colorimetric tests) as well as fasting glucose levels (hexokinase reference method) and insulin levels (automated immunoanalyzer system) to estimate insulin resistance (by HOMA Score) are measured. Samples, which are not analyzed directly, will centrifuged and the plasma aliquots will be frozen at a temperature of −80°. To analyse plasma concentration of the following specific biomarkers blood samples will be defrosted and processed. Cytokine-specific ELISA kits will use to analyse inflammatory serum biomarker interleukin 6 (IL 6), 10 (IL-10), and tumor necrosis factor alpha (TNF-α) according to the manufactures instructions (IL-6/-10: Bender Med-Systems (eBioscience), Austria; TNF-α: Bio-source, USA). The samples will be distributed in duplicates on the plates and the intra-assay and inter-assay coefficient of variation need to be ≤ 10%. High-sensitive c-reactive protein (hsCRP) will be analyzed by immunoturbidimetric latex CRP assay (Cobas 8000, Roche-Diagnostics, Basel).

#### Physical fitness, physical activity, and anthropometry

Physical fitness will be assessed by performing an individualized ramp protocol on the treadmill. We will measure peak oxygen uptake (VO2peak) and maximal heart rate (HRmax). The individualized design with increasing speed and ramp steepness will be used to reach volitional exhaustion after approximately 10 min for every participant. The calculation of this protocol is based on the subject's age and estimated peak metabolic equivalent units (METS) as previously recommended (Bader et al., 1999; Myers and Bellin, 2000). The ECG will be monitored by medical personal during the whole test. Blood pressure will be measured during, immediately after and 3 min after the exercise test. Every minute Ratings of Perceived Exertion (RPE) will be requested (Borg, 1982). Ventilatory parameters, including VO2max and heart rate, will be measured by using the Cortex Metalyzer® 3B metabolic test system (Cortex Biophysik GmbH, Leipzig, Germany).

All participants will get an accelerometer (Aipermon GmbH, Germany) to analyse their physical activity (PA). They have to wear the device on their left hip for the whole day on six consecutive days. Raw data will be copied onto a computer and the data will be viewed using the ActiCoach MPAT2Viewer (Aipermon, GmbH, Germany). The following parameters can be analyzed by this software: total time (minutes per day) spent passively (PAS), actively (ACT), walking (WLK), and fast walking (FWLK). Walking and fast walking times will be joined to a total walking time (TWT). We have previously validated activity modes and accelerometer detection precision (Jehn et al., 2009a,b, 2010, 2011).

Self-reported PA will be measured with the Freiburg Questionnaire of physical activity. The questionnaire interrogates health-related physical activities using self-reported activities within the last week/month. It creates reliable and valid values of PA and inactivity (Frey et al., 1999). The total PA is expressed in hours per week. The Ainsworth Compendium in metabolic equivalents (METS) estimates the intensities. The formula to calculate the energy expenditure per week is described elsewhere (Ainsworth et al., 1993).

Anthropometric data will be measured in a fasting state in the morning. The Inbody 720® (JP Global Markets GmbH, Germany) device will be used to obtain weight, BMI, skeletal muscle mass and body fat (Anderson et al., 2012). Height and waist circumference will be measured by using a normal measuring tape with respect to current guidelines.

#### Potential pitfalls

Our main outcome (cfPWV) as well as most other vascular measurements are affected by age, hemodynamic variability, lifestyle behavior, and circadian fluctuations. To minimize external interference and variability, several measures are considered. Groups are matched for age and sex. Standardized vascular imaging is performed under fasting conditions in the morning after 10 min of rest in a supine position. Participants are encouraged to refrain from alcohol, caffeine and if applicable from smoking 12 h prior to the examinations. Exercise and vigorous physical activity should not be performed 24 h before the visits. The OSR group is allowed to take their medication in the morning. These measures help to minimize intra-individual as well as within-day variability.

Change of medication or start of a structured diet during the 12 week-week intervention or control condition are indications for exclusion. In addition, the start of structured exercise in the control group is considered an exclusion criterion. To prevent the start of exercise in the control group, all participants of this group are invited to take part in the voluntary supervised exercise program after the post measurements. To prevent high drop-out rates and optimize adherence to the exercise regime, every exercise session starts with strength, mobilization and coordination exercises and includes a warm-up and a cool-down. The first 2 weeks of the exercise program focus on technical and coordinative skills at lower intensities before HIIT is started in the third week into the program.

## Discussion and conclusion

**Figure 3:**
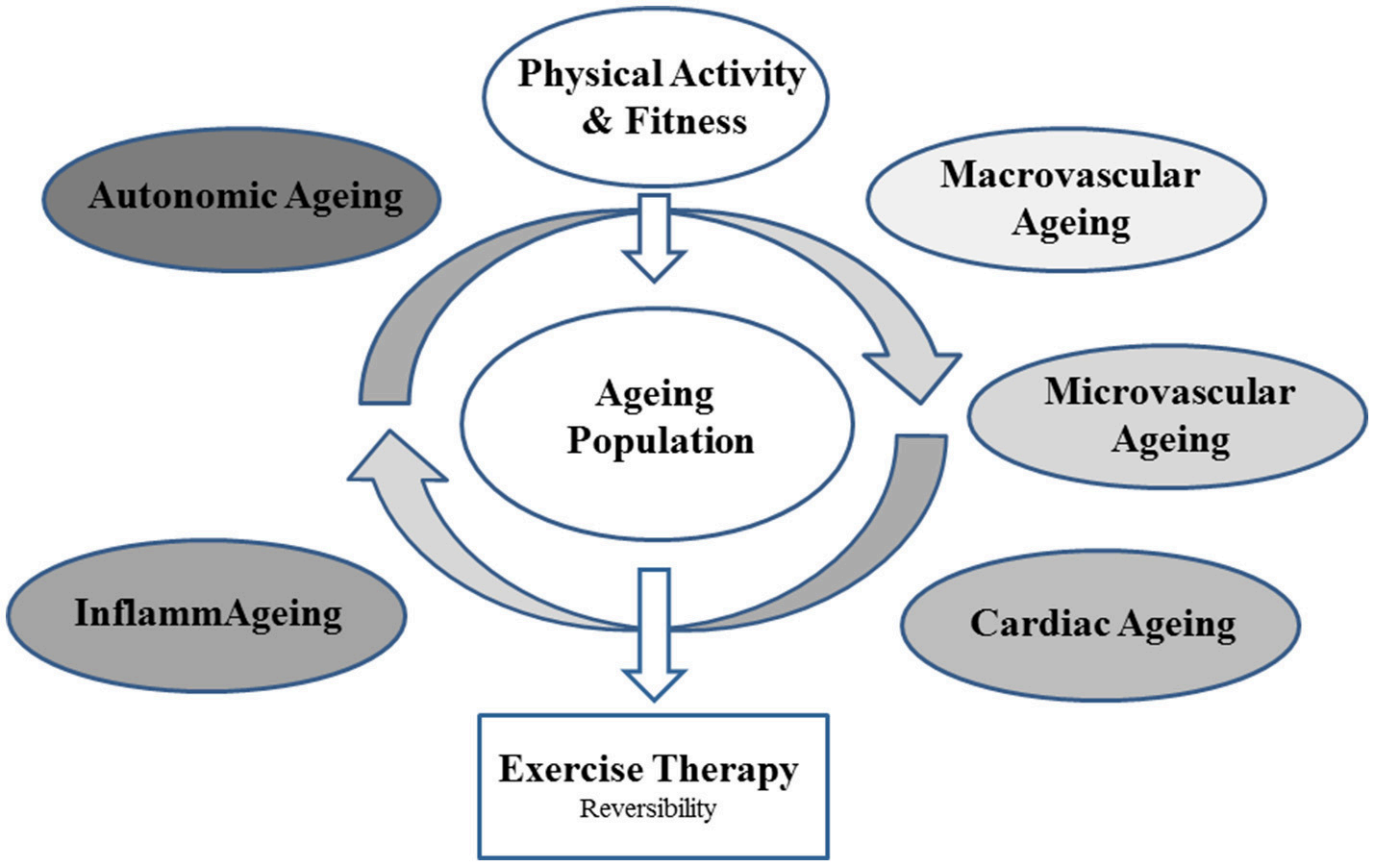
Principle concept of the systems physiology approach.

The study design offers a concept for a systems physiology approach on the mechanisms of aging and the role of physical activity and fitness. The cross-sectional approach enables to investigate the association of physical activity with the process of normal, healthy aging comparing the HOA group with HOS. In addition, the impact of cardiovascular disease on physiological functioning is examined by comparing the HOS group with OSR. The reversibility of pathophysiological aging in patients with cardiovascular risk is explored by applying an interventional exercise treatment in the OSR group. Our study concept investigates the interplay of physiological systems and how these are affected by physical activity and fitness. To what extent can physical fitness prevent or delay physiological dysfunction? The study aims at clarifying some of the main mechanisms by which exercise improves the process of aging. The multimodal concept of the study is depicted in Figure 3.

The ExAMIN AGE Study resembles new approaches in the structural and functional diagnostics of aging processes including systems such as the vasculature, the heart, autonomic function and inflammation. The results have a focus on vascular imaging and on the non-pharmaceutical treatment of vascular health in older inactive individuals at risk. The integrative approach will help to determine the best non-invasive means to diagnose and define cardiovascular risk in advanced age and disease. This study design allows the examination of pathophysiological links between small and large arteries, the so called “vascular coupling” or “arterial cross-talk.” To date little is known about the interaction between the two vascular beds and how they affect each other. It also enables to examine the cross-talk between the vasculature and the heart. Exercise interventions can improve both entities and play a key role in the treatment of cardiovascular and metabolic diseases.

Our study has some limitations. We are aware that the recruitment of healthy older sedentary individuals (HOS) is challenging. It needs to be ascertained that all three groups are strictly matched for age. The analysis of the association of physical fitness with cardiovascular health and aging processes in the three groups represents a cross-sectional approach and is not based on prospective longitudinal data. Life-long activity is analyzed by subjective reporting but is supported by objective analysis using accelerometry and spiroergometry. The cross-sectional study design does not give evidence of a temporal relationship between physical activity exposure and physiological outcome. However, the interventional part of the study, where participants are randomly allocated to the intervention or the control group, allows for a causal and temporal analysis of the improvement of aging processes in individuals at risk. The extensive phenotyping of vascular health and other physiological systems as well as the analysis of the impact of HIIT are the main strengths and innovations of the study.

In conclusion, the concept links cardiovascular prevention and exercise medicine in a systems physiology approach. It aims to help define new recommendations for treatment guidance of exercise therapy in an aging population. We aim to demonstrate the importance of specific physical activity programs for seniors to achieve healthier aging as a long-term goal. Amelioration of vascular function represents improvement of disease- and age-related end organ damage and best describes the potential to contain vascular health. The study will generate results which will help to understand better the mechanisms of vascular aging and the interplay with other physiological systems. The study approach has the potential for transfer in other age groups and clinical settings.

## Ethics statement

This study was carried out in accordance with the recommendations of the Helsinki Declaration and the SPIRIT Guidelines. All subjects gave written informed consent in accordance with the Declaration of Helsinki. The protocol was approved by the Ethics Committee of Northwest and Central Switzerland (EKNZ 2015-351).

## Author contributions

LS: drafted the manuscript, performed examinations, is responsible for general data collection and analysis of retinal vessels and cardio respiratory fitness, organized and performed the exercise interventions; AD: helped draft the manuscript, performed the large artery measurements as well as cardiac imaging, medical examination, and supervision of all participants; JS: performed the sample size estimation and was responsible for statistical considerations; AS-T: participated in the study design and helped draft the manuscript; HH: designed the study and is principal investigator, wrote the manuscript and helped analyse the data. All authors read and approved the final manuscript.

### Conflict of interest statement

JS has been an employee of F. Hoffmann-La Roche Ltd since December 1, 2016. The present study was conducted before JS joined F. Hoffmann-La Roche Ltd and has no connection to her employment by the company. The other authors declare that the research was conducted in the absence of any commercial or financial relationships that could be construed as a potential conflict of interest.

## Abbreviations

HOA: healthy older Active
HOS: healthy older sedentary
OSR: older sedentary at risk
HIIT: high intensity interval training
PA: physical activity
VO_2_max: maximal oxygen uptake as a measure of physical fitness
MET: metabolic equivalent
CVD: cardiovascular disease
PWV: pulse wave velocity
cfPWV: carotis-femoral PWV
ftPWV: femoral tibial PWV
AIx: augmentation index
AIx@75: AIx at heart rate 75/min
cPP: central pulse pressure
IMT: intima media thickness
HRV: heart rate variability
SVA: static retinal vessel analysis
DVA: dynamic retinal vessel analysis
CRAE: central retinal equivalent
CRVE: central retinal equivalent
AVR: arteriolar-to-venular diameter ratio.
